# Expression and Prognostic Role of CXCL1 Gene in Colorectal Adenocarcinoma

**DOI:** 10.1155/2022/5504731

**Published:** 2022-08-02

**Authors:** Chao Xia, Lifeng He, Yi Sun

**Affiliations:** Gastrointestinal Surgery Affiliated Xiaoshan Hospital, Hangzhou Normal University, Zhejiang, Hangzhou 311200, China

## Abstract

In this manuscript, we have extensively examined expression and prognosis of CXCL1 gene in colorectal adenocarcinoma (COAD) using different cases of colorectal adenocarcinoma and tissues. To verify this, protein and mRNA expressions of cxcl1 were identified through RT-PCR and immunohistochemistry in 30 cases of colorectal adenocarcinoma and adjacent tissues, which were surgically resected from January to July 2021 in our hospital, and relationship between CXCL1 mRNA and clinicopathological features and protein expression was analyzed. CXCL 1 mRNA in COAD carcinoma's expression was considerably higher than in the adjacent normal intestine. At the same time, CXCL 1 diagnostic receiver operating characteristic (ROC) curve had preferably higher value of the diagnostic for area under curve (AUC) = 0.912, 95%, COAD (*P* < 0.001, CI = 0.825–0.969). We have observed that CXCL1 gene was closely linked with preoperative CEA level (*P*=0.007) and gross tumor typing (*P*=0.039). Finally, we have concluded that that CXCL1 can be a possible biomarker for stress prognosis and diagnosis.

## 1. Introduction

Colorectal cancer (CRC) is a kind of cancer that affects the colon and rectum. CRC is one of the cancers with the highest rates of morbidity and death worldwide. According to data, approximately 1.8 million cases of the CRC and around 860,000 deaths are reported in the year 2018, which is approximately 10% of the overall cases of cancer and fatalities. CRC is considered as 3^rd^ common malignancy and in terms of death ratio it is second [1]. CRC is one of China's top five malignancies in terms of morbidity and fatality [2]. In CRC patients, cancer metastasis is still the major cause of mortality. Patients have primary CRC and an 80–90 5-year overall rate of survival; however, patients with metastatic CRC have a 5-year rate of the survival of just 50 percent as low as 5–10 [3, 4]. CRC, like many other diseases, preferably cancers are assumed as a disease of type heterogeneous, in which tumor formation, progression, and metastasis are linked to genetic diversity, cellular environment, and external environmental effects [5].

CRC has a high incidence and fatality rate, and surgical resection is currently the most common therapeutic option [6]. The earlier the CRC therapy begins, the better the outcome; early-stage cancer has a 5-fold survival rate compared to advanced disease [7]. Patients with tumor infiltration into the lamina propria (TNM stage TIS, N0, MO) had a 100 percent 5-year survival rate, but patients with invasive T1 (submucosal) or T2 (muscular) malignancy had a 90 percent 5-year survival rate. Those who were more aggressive but did not include lymph nodes (t3-4, N0, MO) had a 5-year survival rate of around 70%, whereas those who were regional lymph node positive had a 5-year survival rate of about 40% (any *T*, N1-3, MO). The 5-year survival rate for patients with distant metastases (any *T*, any N, M1) is around 5% [8]. Patients with successfully operated stage I cancer had a 5-year recurrence rate of 5%, 13% for stage II, and 33% for stage III, with recurrence rates varying from 9 to 22 percent for stage II to 17 to 44 percent for stage III, depending on the number of risk factors [9]. As a result, early detection and treatment of CRC are critical for the prognosis of patients.

CXC motif chemokine ligand 1 (CXCL1) is a tiny cytokine that belongs to the CXC chemokine family [10]. It was formerly identified as the Gro1 oncogene. Human melanoma cells release CXCL1, which has mitogenic characteristics and is linked to melanoma aetiology [11, 12]. CXCL1 is a neutrophil chemoattractant that is produced by the cells of neutrophils, macrophages, and epithelial [13, 14]. CXCL1 is implicated in angiogenesis, inflammation, wound healing, and cancer, as well as blocking the oligodendrocyte precursor's migration in the spinal cord. CXCL1 gene is located on human chromosome 4 and is the gene of other CXC chemokines. Preliminary studies in mice have shown that CXCL1 can reduce the severity of multiple sclerosis and may provide neuroprotective function [15]. CXCL1 expression in colon cancer is much greater than in normal colon tissue, according to previous research. In metastatic CRC, high CXCL1 expression is a poor prognostic indicator; CXCL1 expression is linked to a poor prognosis in stage III CRC [16]. As a result, the role of CXCL1 in the diagnosis and prognosis of colonic adenocarcinoma was investigated in this study (COAD).

In this manuscript, we have extensively examined expression and prognosis of CXCL1 gene in colorectal adenocarcinoma (COAD) using different cases of colorectal adenocarcinoma and tissues. To verify this, protein and mRNA expressions of cxcl1 were identified through RT-PCR and immunohistochemistry in 30 cases of colorectal adenocarcinoma and adjacent tissues, which were surgically resected from January to July 2021 in our hospital, and relationship between CXCL1mRNA and clinicopathological features and protein expression was analyzed. CXCL 1 mRNA in COAD carcinoma's expression was considerably higher than in the adjacent normal intestine. At the same time, CXCL 1 diagnostic ROC curve had preferably higher value of the diagnostic for AUC = 0.912, 95%, COAD (*P* < 0.001, CI = 0.825–0.969).

The rest of the paper is organized as follows.

In the subsequent section, the proposed evaluation mechanism is described in detail, where it is depicted clearly how various participants were selected and how the evaluation was carried out. In section 3, numerous results of the proposed scheme in terms of different evaluation metrics were presented along with possible justification and analysis. Finally, concluding remarks are given.

## 2. Methods

In this section, we are going to describe a detailed explanation of the proposed mechanism along with the participants.

### 2.1. Participants in the Evaluation Process

The surgically resected cancer tissue and normal adjacent colon tissue were collected from patients with colorectal adenocarcinoma in the department of colorectal and anal surgery in our hospital from January to July 2020. All patients did not receive radiotherapy or chemotherapy before operation. After operation, they were pathologically diagnosed as spinal cord injury and signed the informed consent.

### 2.2. Information Collection

Basic information and clinicopathological factors of patients were collected, including gender, age, preoperative carcinoembryonic antigen (CEA), tumor/lymph node/metastasis (TNM) stage, tumor site, tumor gross type, tumor thrombosis, tumor size, number of tumors, positive lymph nodes, radical resection, tumor metastases, nerve invasion, and postoperative chemotherapy which were all factors to consider. The American Joint Commission on Cancer (AJCC) Lymph Node Metastasis Staging System (8th edition, 2017) [16] was used to identify and classify TNM staging. It is divided into left and right semicolon colons based on anatomical sites. The left semicolon colon includes the colon in the splenic flexure, descending colon, and sigmoid colon, while the right semicolon colon includes the colon in the cecum, ascending colon, hepatic flexure, and transverse colon [17]. Colectomy and regional lymph node dissection were the major surgical methods used.

### 2.3. Tissue Specimen Collection

After the tissue was surgically removed, the tumor about the size of soybeans and the normal colon tissue more than 3 cm adjacent to the tumor were cut out with ophthalmic scissors (note: the instruments for cancer tissue and normal colon tissue adjacent to the tumor were used separately), cut into pieces, and placed into a 1.5 ml EP tube. RNA protection solution ((1 ml) was added to completely soak the tissue. Place the EP tube in a 4°C refrigerator test-tube rack overnight (allowing the RNA protection solution to mix well with the tissue). Transfer the EP tube to an −80°C refrigerator on the second day. The EP tube body is marked with the patient's name, hospitalization number, and operation date (collection date), and the EP tube cap is marked with the patient's hospitalization number. Each patient received 2 tubes of cancer and 2 tubes of adjacent normal colon tissue.

### 2.4. Extraction, Purification, and Identification of Tissue Total RNA

Total RNA was extracted from tissues in accordance with the instructions of Trizol. 2 *μ*L of RNA sample was taken, and the absorbance of RNA at 260 nm was measured by micro-UV spectrophotometer Q5000. The absorbance A260 and A280 of RNA at 260 nm and 280 nm was measured for quantitative RNA purity, and OD260/OD280 was recorded. The ratio of all samples was required to be 1.7 < OD260/OD280 < 2.0, lower than this value indicated that it contained protein impurities. Repurify it with chloroform. RNA integrity was determined by agarose gel electrophoresis.

### 2.5. Reverse Transcription

Total RNA was used as template to synthesize cDNA using invitrogen reverse transcription kit. Take a 0.2 ml DEPC-treated PCR tubes in which the reverse transcription reaction solution was prepared in an ice bath.

Reverse transcription reaction was performed according to the instructions of the reverse transcription kit. In the PCR tube, 1 *μ* of reverse transcription product, 0.25 *μ*L of upstream and downstream primers, PCR reaction mix 12.5 *μ*L, then ddH2O supplement volume to 25 *μ*L. After mixing, the samples were immediately placed in PCR apparatus for reaction according to the following procedures. After the reaction, the samples were stored at −20°C for later use. Reaction conditions are 95°C for 2 min, 95°C for 15 s, 62°C for 30 s, and a total of 35 cycles. All primers were synthesized by Shanghai Shenggong Co., LTD. The upstream sequence of the for internal reference in Table 1. The detection of different genes in the same sample should be carried out in different PCR tubes. After amplification, the amplification curve and the melting curve were analyzed to obtain the cycle threshold (CT value). Finally, the differential multiple of CXCL1 expression in tumor tissues relative to normal tissues was calculated by 2^−ΔΔCT^ method.

### 2.6. Statistical Analysis

The continuous data of the normal distribution were represented by Mean SD, and the continuous variables of the non-normal distribution were converted into dichotomy variables according to the median as the cut-off point. Chi-square test was used to analyze the correlation between clinical factors and CXCL1. And only normal distribution data can use *t*-test.

## 3. Results and Evaluation

In this section, a detailed analysis of various experimental results, specifically in terms of numerous evaluation metrics, is presented. For an easy follow-up, the proposed scheme is described with both discussion and experimental results.

### 3.1. Detection of CXCL1 Expression in COAD Patients and Adjacent Normal Colon Tissues

**Figure 1:**
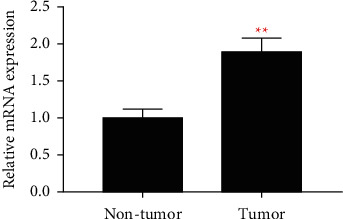
CXCL1 expression in cancer and adjacent normal colon tissues of COAD patients.

The study comprised thirty COAD patients, 20 men, and 10 women, with a median age of 58.75 years (range 34 to 80 years). RT-qPCR was used to identify real-time CXCLI gene mRNA in 30 COAD patients (Figure 1). CXCL 1 mRNA expression in COAD cancer tissues was considerably greater than in nearby normal intestinal tissues, according to a paired *T* test. Simultaneously, the CXCL 1 diagnostic ROC curve revealed a greater diagnostic value for COAD (P 0.001, AUC = 0.912, 95 percent CI = 0.825–0.969).

### 3.2. CXCL1 Expression in Cancer and Adjacent Normal Colon Tissues of COAD Patients

We grouped CXCL1 gene mRNA expression levels in cancer tissues of COAD patients into groups and calculated the relationship between CXCLI gene mRNA expression and clinical factors of patients (Table 2). The results showed that CXCL1 gene was correlated with preoperative CEA level (*P*=0.007) and gross tumor typing (*P*=0.039).

## 4. Discussion

Colon cancer is one of the common malignant tumors. 60% of patients have clinical and pathological evidence of liver metastasis. Liver metastasis of colon cancer shows obvious organophilic, and the rates of simultaneous and isochronous liver metastasis are 43.7% and 56.3%, respectively [16]. This organophilic is a manifestation of directional migration of tumor cells. The “homing” theory holds that different organs have the special ability to capture or attract specific types of tumor cells by secreting chemokines. It has been confirmed that the directional metastasis process of some tumor cells shows similar characteristics to leukocyte chemotactic migration [17].

At present, surgical resection is still the main means of CRC treatment. The earlier the CRC is treated the better with survival rates about five times higher than for advanced cancer. So, early diagnosis and early treatment of colon cancer are particularly important for the prognosis of patients. At the time of initial diagnosis of CRC, only 15% of stage I patients were diagnosed, and most patients were already in the middle and late stage. Although colonoscopy is still the gold standard for detecting colon cancer, it is invasive, costly, and inconvenient, making it unsuitable for routine screening. Good tumor indicators have a high sensitivity and specificity for tumor diagnosis, and they help to detect malignancies early. Previous research on biological markers for colon cancer has had mixed findings. For colon cancer diagnosis and postoperative follow-up, serum carcinoembryonic antigen (CEA) is a useful tumor marker. Serum CEA level is positively correlated with tumor node metastasis (TNM) stage. The positive rates of serum CEA in TNM stage I, II, III, and IV patients were about 25%, 45%, 75%, and 85%, respectively. CEA has little significance for the early diagnosis of colon cancer. Therefore, to find more sensitive, more specific, and easier to operate biological markers is the current research focus.

In the study on the diagnostic value of CXCL1 in CRC, Wen et al. confirmed that the expression of CXCL1 in CRC was higher than that in normal colonic epithelium by real-time PCR and immunohistochemistry [18]. Sipos et al. found that on the basis of tissue microarray analysis, the matrix expression of MMP3 and CXCL1 can correctly distinguish high-grade dysplastic sessile adenoma stage and early CRC [19]. In the study of the role of CXCL1 on the occurrence and development of CRC, Ogata et al. [20]. detected Groalpha immunohistochemistry in 62 primary CRC specimens and discussed the relationship between Groalpha expression and clinicopathological features; it was found that the expression of Groalpha was significantly correlated with tumor size, tumor stage, depth of invasion, lymph node metastasis, and survival rate. Wang et al. found that CXCL1 released by cancer cells induced microvascular endothelial cell migration and tubulation *in vitro*. In addition, prostaglandin E2 promotes tumor growth *in vivo* by inducing CXCL1 expression, resulting in increased tumor angiogenesis [21]. In the study of Hsu et al., transcriptome analysis of CXCL1 treated SW620 cells showed that CXCL1 could increase the expression of potential oncogenes in colon cancer. Analysis of public data showed that CXCL1 driven oncogenes and mir-105 had a negative impact on the prognosis of colon cancer [22]. Le Rolle et al. suggested that high CXCL1 expression is a biomarker of poor prognosis in metastatic CRC [23]. Moreover, silencing CXCL1 inhibited the tumorigenic growth of KRAS mutant CRC cells. In the study of Cai et al., adiponectin partially promotes the aging of stromal cells in invasive colon cancer by producing CXCL1 and may be used as a therapeutic target for tumor patients [24].

Therefore, this study shows that CXCL1 may be a potential biomarker for the diagnosis and prognosis of load. This study verifies previous studies from the gene level. In terms of diagnostic value, the mRNA expression of CXCL1 in COAD cancer tissues is significantly higher than that in adjacent normal colon tissues, and ROC data show that CXCL1 does not significantly affect the prognosis of COAD. Because the number of patients included in this study is too small, there are certain limitations, and the research method is too single, and the reliability of the results needs to be further verified.

## 5. Conclusion

In this manuscript, we have extensively examined expression and prognosis of CXCL1 gene in colorectal adenocarcinoma (COAD) using different cases of colorectal adenocarcinoma and tissues. To verify this, protein and mRNA expressions of cxcl1 were identified through RT-PCR and immunohistochemistry in 30 cases of colorectal adenocarcinoma and adjacent tissues, which were surgically resected from January to July 2021 in our hospital, and relationship between CXCL1mRNA and clinicopathological features and protein expression was analyzed. CXCL 1 mRNA in COAD carcinoma's expression was considerably higher than in the adjacent normal intestine. At the same time, CXCL 1 diagnostic ROC curve had preferably higher value of the diagnostic for AUC = 0.912, 95%, COAD (*P* < 0.001, CI = 0.825–0.969). Experimental results have verified the exceptional performance of the proposed scheme in terms of various performance evaluation metrics.

In future, the proposed methodology has the capacity to be extended further by integrating it with existing state of the art approaches to form a hybrid and more useful approach.

## Figures and Tables

**Table 1:** The upstream sequence of the primer for internal reference.

Name	Primer	Length
GAPDH	5′-CGGGAAATCGTGCGTGA-3′	116 bp
	5′-TCAGGCAGCTCGTAGCTCTT-3′	

CXCL1	5′-CAAACCGAAGtCATAGCCACA-3′	120 bp
	5′-CTCCTAAGCGATgCTCAAACA-3′	

**Table 2:** CXCL1 expression in cancer and adjacent normal colon tissues of COAD patients.

Variable		Low	High	X^2^	*P*
Gender					1.00
	Male	11	9		
	Female	5	5		

Age					1.00
	<65	11	9		
	>65	5	5		

CEA (ng/ml)					0.007
	1–5	11	13		
	>5	5	1		

TNM stage				2.576	0.517
	I	1	2		
	II	8	10		
	III	3	2		
	IV	2	1		

Location					1.00
	Right	3	3		
	Left	12	12		

Tumor type				6.782	0.038
	Invasive	1	2		
	Ulcerative	14	6		
	Mass	2	5		

Differentiation					0.682
	Well	13	14		
	Poor	2	1		

Tumor thrombus					0.702
	No	11	16		
	Yes	3	1		

Tumor size					1.00
	<5	3	2		
	>5	12	13		

Lymph node					0.148
	Negative	8	16		
	Positive	4	2		

Tumor transfer					0.622
	No	10	14		
	Yes	4	2		

Nerve infiltration					1.000
	No	13	13		
	Yes	2	2		

## Data Availability

The datasets used and analyzed during the current study are available from the corresponding author upon reasonable request.
